# A Hydrogel-Based Epirubicin Delivery System for Intravesical Chemotherapy

**DOI:** 10.3390/molecules21060712

**Published:** 2016-06-01

**Authors:** Ching-Wen Liu, Yu-Tse Wu, Kai-Jen Lin, Tsan-Jung Yu, Yu-Liang Kuo, Li-Ching Chang

**Affiliations:** 1 School of Pharmacy, Kaohsiung Medical University, No.100, Shih-Chuan 1st Road, Sanmin District, Kaohsiung 807, Taiwan; fruit0227@hotmail.com; 2 Department of Pathology, E-Da Hospital, I-Shou University, No.1, Yida Road, Yanchao District, Kaohsiung 824, Taiwan; ed102328@edah.org.tw; 3 Department of Urology, E-Da Hospital, I-Shou University, No.1, Yida Road, Yanchao District, Kaohsiung 824, Taiwan; ed100622@edah.org.tw; 4 Department of Medical Imaging and Radiological Sciences, Chung Shan Medical University, No.110, Sec. 1, Jianguo North Rd., South District., Taichung 402, Taiwan; yuliangkuo622@gmail.com; 5 Department of Occupational Therapy, I-Shou University, No.8, Yida Road, Yanchao District, Kaohsiung 824, Taiwan; 6 Department of Pharmacy, E-Da Hospital, I-Shou University, No.1, Yida Road, Yanchao District, Kaohsiung 824, Taiwan

**Keywords:** epirubicin, hydrogel, intravesical, bladder cancer

## Abstract

This study aimed to examine the efficacy of epirubicin-loaded gelatin hydrogel (EPI-H) in the treatment of superficial urothelium carcinoma. Hydrogel was prepared by Schiff base-crosslinking of gelatin with glutaraldehyde. EPI-H exhibited high entrapment efficiency (59.87% ± 0.51%). EPI-H also increased epirubicin accumulation in AY-27 cells when compared with the effect of aqueous solutions of epirubicin (EPI-AQ); respective epirubicin-positive cell counts were 69.0% ± 7.6% and 38.3% ± 5.8%. EPI-H also exhibited greater cytotoxicity against AY-27 cells than that of EPI-AQ; IC_50_ values were 13.1 ± 1.1 and 7.5 ± 0.3 μg/mL, respectively. Cystometrograms showed that EPI-H reduced peak micturition, threshold pressures, and micturition duration, and that it increased bladder compliance more so than EPI-AQ. EPI-H enhanced epirubicin penetration into basal cells of urothelium *in vivo*, whereas EPI-AQ did so only to the umbrella cells. EPI-H inhibited tumor growth upon intravesical instillation to tumor-bearing bladder of F344 rats, inducing higher levels of caspase-3 expression than that observed with EPI-AQ treatment; the number of caspase-3 positive cells in treated urothelium carcinoma was 13.9% ± 4.0% (EPI-AQ) and 34.1% ± 1.0%, (EPI-H). EPI-H has value as an improved means to administer epirubicin in intravesical instillation treatments for bladder cancer.

## 1. Introduction

The standard-of-care therapy for non-muscle-invading bladder cancer (NMIBC) is transurethral resection of bladder tumor (TURBT) followed immediately by a single intravesical dose of a chemotherapeutic agent (e.g., epirubicin) or immunotherapeutic agent (e.g., BCG) to reduce recurrence and progression to a muscle-invasive disease [1]. Intravesical delivery provides regional therapy in which drugs are directly administered into the bladder through a urethral catheter [2]. Intravesical therapy has advantages in allowing more targeted delivery of therapeutic agents to tumor cells located in the bladder epithelium and in reducing the side effects associated with systemic drug absorption. Overall, this enables a broader therapeutic window. However, two major physiological hurdles restrict intravesical efficacy, and this reduced efficacy can result in a high cancer recurrence rate. The first hurdle is limited dwell time of a drug in the bladder (2 h) due to washout during voiding. The second hurdle is the limited uptake of drug into the urothelial cells (normal or malignant) due to the unique permeability barrier properties of the urothelium [3].

The current formulation of epirubicin to be instilled in the bladder is a lyophilized powder that is dissolved in normal saline. This formulation is typically administered in episodes of 2-h durations. Irritation to the urothelium, urine washout, and watertight urothelium barriers prevent this aqueous epirubicin solution from achieving sustained exposure to the urothelium. The 1- and 2-year cancer recurrence-free rates after initial TURBT are 25% and 12.5%, respectively, and the recurrence-free rates when epirubicin treatment is applied after TUR are 58.5% and 38.6%, respectively [4]. Given the high recurrence rates, we speculate that a scaffold for optimizing epirubicin release to the luminal bladder may improve drug efficacy. 

Strategies for improving intravesical therapy include physical and chemical approaches to increasing both drug penetration and drug residence time in the bladder [5,6]. We previously constructed poly(ethyl-2-cyanoacrylate) (PECA) epirubicin-loaded nanoparticles for enhancing intravesical delivery of epirubicin [7]. We have since then developed gelatin-based muco-adhesive nanocomposites as intravesical delivery scaffolds for gene therapy applications [8]. Gelatin-based hydrogels provide good viscosity for transduction into the bladder through a urethral tube. They also exhibit urothelium adhesion, thus enhancing urothelial penetration of drugs associated with the hydrogel. Here, the present study evaluated the feasibility of using hydrogel to provide an intravesical scaffold for applications of epirubicin in the chemotherapy of NMIBC. 

We are the first to propose the application of epirubicin-loaded gelatin hydrogel carriers for intravesical usage and the first to study its feasibility in therapies targeting bladder tumors. Prior to the biological applications described in this study, we fully characterized the appearance of epirubicin-loaded 15% gelatin hydrogel (denoted as EPI-H). This characterization included an evaluation of fine structure by scanning electron microscopy (SEM). The cytotoxicity of epirubicin-loaded gelatin against the AY-27 bladder cancer cell line was determined *in vitro*. The potential for enhanced apoptosis after EPI-H treatment was evaluated by means of double-labeling with FITC-tagged annexin V and propidium iodide. Apoptosis was also assessed through the cleaved/pro caspase-3 ratio as determined by western blotting. The adhesion of EPI-H to F344 rat bladder urothelium and penetration of epirubicin through the urothelium were assessed using fluorescent microscopy; histological features of the urothelium were subsequently inspected. Urodynamic parameters assessed after administration of EPI-H included peak micturition pressure, threshold pressure, micturition duration, and bladder compliance. Finally, the efficacy of EPI-H against bladder tumors formed by AY-27 cells in F344 rats was assessed. 

## 2. Results

### 2.1. Characterization of EPI-H

**Figure 1:**
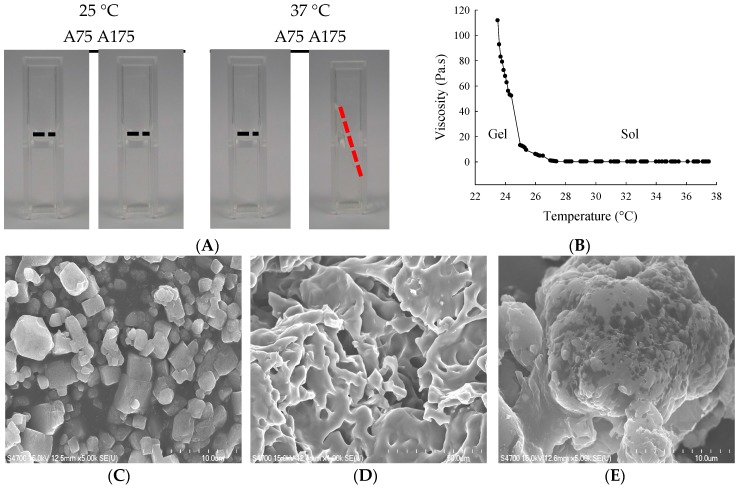
(**A**) Visual inspection of gelatin hydrogel at 25 °C or 37 °C; (**B**) Viscosity (Pa.s) *vs.* temperature for gelatin A175 hydrogel. Scanning electron microscopy (SEM) images showing microstructures of (**C**) epirubicin; (**D**) gelatin A175 hydrogel (H); and (**E**) epirubicin-loaded hydrogel (EPI-H). Magnification: (b,d) ×5000, (c) ×1000.

We first characterized the impact of gelatin type (A75 or A175) on hydrogel yield, hydration ratio, size, viscosity, and torque (Table 1). For 15% gelatin type A75 and A175 hydrogel, mean yields were 89.7% and 98.0%, mean hydration ratios were 86.8% and 87.2%, mean particle sizes were 183.6 and 124.6 nm, mean viscosities were 32.2 and 78.7 Pa.s., and mean torques were 56.7% and 67.0%, respectively. The thermosensitivities of 15% gelatin A75 and A175 hydrogel were examined by visual inspection (fluidity and turbid) at 25 °C and 37 °C (Figure 1A). The 15% gelatin A175 hydrogel showed high viscosity at 25 °C and became fluid at 37 °C. The influence of temperature changes on gelatin A175 hydrogel viscosity values is shown in Figure 1B. This optimal fluidity made it easy to administer, providing good flow, coverage, and adhesion to the urothelium in the bladder cavity. Thus, the 15% gelatin A175 (denoted as H), which afforded satisfactory yield rate, hydration ratio, viscosity, and thermosensitive fluidity, was selected as the carrier for epirubicin in the biological evaluations conducted in this study. 

The entrapment efficiency of epirubicin (0.25 mg/mL) in 15% gelatin A175 was 59.87% ± 0.51%. The SEM photographs showed epirubicin as a fine irregular crystal structure (Figure 1C). The lyophilized 15% gelatin A175 hydrogel showed fibrous details (Figure 1D). When epirubicin was loaded into the hydrogel, SEM showed the epirubicin to be partially dispersed, and that it formed a dense and conglomerate structure (Figure 1E).

### 2.2. In Vitro Epirubicin Accumulation and Cytotoxicity

**Figure 2:**
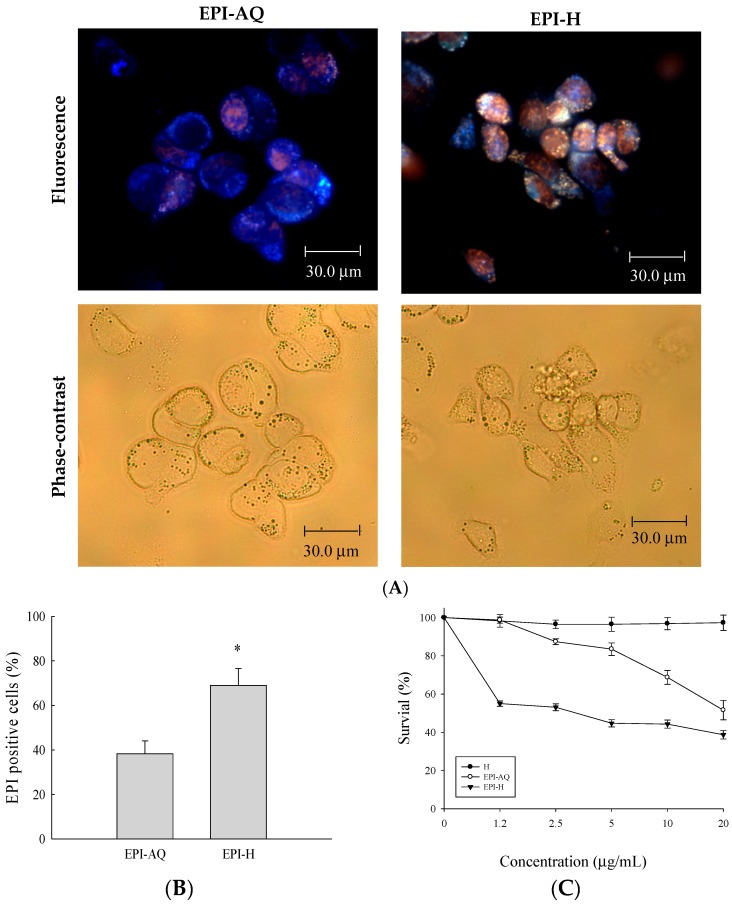
Epirubicin accumulated in AY-27 cells and induced cytotoxicity. (**A**) Fluorescent images of epirubicin (red) and DAPI (nuclei, blue), with phase-contrast images of AY-27 cells after EPI-AQ and EPI-H treatment for 18 h. (magnification, ×400) (**B**) The statistics of epirubicin-positive cells in the EPI-AQ and EPI-H treatment AY-27 cells; (**C**) The cytotoxicity of AY-27 cells with H, EPI-AQ, and EPI-H treatment for 18 h prior to the MTS assay. All results are expressed as the mean ± standard error of four independent experiments. * *p* < 0.05 in cases of EPI-H versus EPI-AQ.

Epirubicin (0.25 mg/mL) was dissolved in normal saline (EPI-AQ) or dispersed in hydrogel (EPI-H). Treatment with EPI-H allowed greater epirubicin accumulation in AY-27 cells than that allowed by treatment with EPI-AQ (Figure 2A). The epirubicin-positive cell counts from EPI-AQ and EPI-H treatments of AY-27 were 38.3% ± 5.8% and 69.0% ± 7.6%, respectively (Figure 2B). As shown in Figure 2C, the half-maximal inhibitory concentrations (IC_50_ values) of hydrogel (H), EPI-AQ, and EPI-H in AY-27 cells were 196.2 ± 0.7, 13.1 ± 1.1, and 7.5 ± 0.3 μg/mL, respectively. EPI-H exhibited significantly increased cytotoxicity against AY-27 cells when compared with the effect of EPI-AQ (*p* = 0.013). The lack of cytotoxicity associated with the hydrogel (H) confirmed that it could be safely used in intravesical applications.

### 2.3. Enhanced Apoptosis by Caspase-3 Pathway

**Figure 3:**
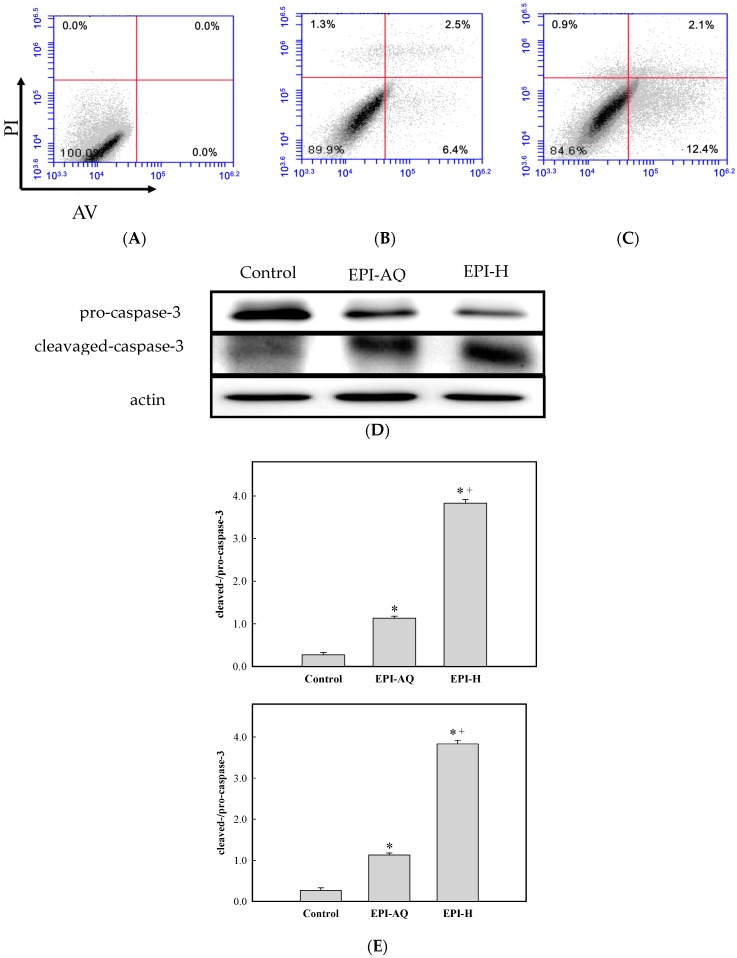
EPI-H enhanced apoptosis in AY-27 cells through activation of caspase 3 pathway. (**A**) AY-27 cells were treated with (**B**) EPI-AQ and (**C**) EPI-H then double-stained with Annexin V-FITC (AV) and PI followed by flow cytometry analysis. Data are presented as a percentage of the cell population; (**D**) As shown by western blot analysis EPI-AQ and EPI-H induce conversion of pro-caspase 3 to cleaved-caspase-3 in AY-27 cells. (**E**) Histogram showing the ratios of cleaved-/pro-caspase-3 in control, EPI-AQ-, and EPI-H-treated cells. Results are expressed as the mean ± SD. * *p* < 0.05 in cases of EPI-AQ and EPI-H *vs.* control. ^+^ *p* < 0.05 for EPI-H *vs.* EPI-AQ.

The apoptotic effect of EPI-H was examined by Annexin V-FITC (AV) and propidium iodide (PI) double staining, and by fluorescence-activated cell sorting (FACS). AV-positive/PI-negative cells were scored as early apoptotic, and AV-positive/PI-positive cells were scored as late apoptotic cells. As shown in Figure 3A,C, the percentages of AY-27 cells in the early/late apoptotic stage observed after control, EPI-AQ, and EPI-H treatments were 0.0%/0.0%, 6.4%/2.5%, 12.4%/2.1%, respectively. In other words, EPI-H treatment induced higher numbers of AY-27 cells to enter early apoptosis.

Pro- and cleaved-caspase-3 proteins were measured by western blotting to assess apoptotic rates Figure 3D shows that EPI-H treatment caused a reduction in pro-caspase-3 levels and a concomitant increase in levels of cleaved-caspase-3 in Ay-27 cells. Figure 3E shows that mean ratios of cleaved-/pro-caspase-3 levels associated with control, EPI-AQ, and EPI-H treatments were 0.27, 1.13, and 3.83, respectively. These results indicated that the enhanced apoptosis induced by EPI-H in AY-27 cells occurred through the caspase activation pathway.

### 2.4. Urodynamic Parameters

**Figure 4:**
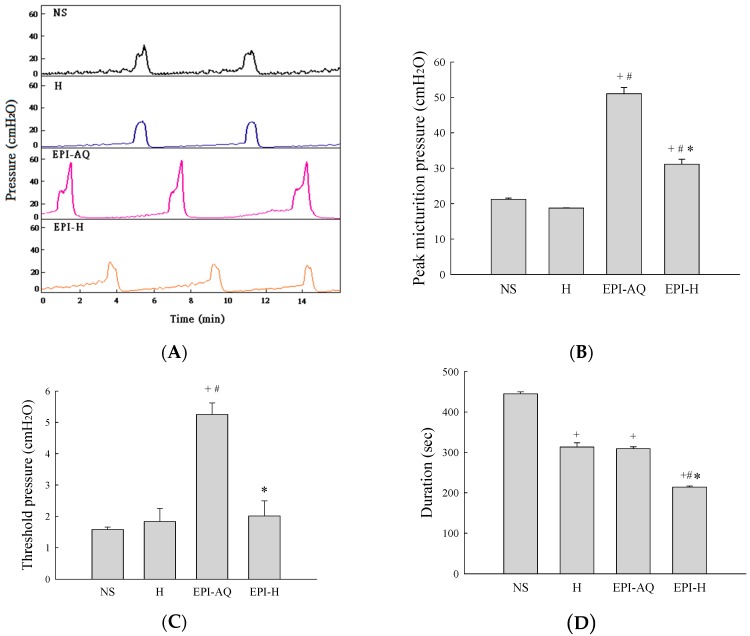

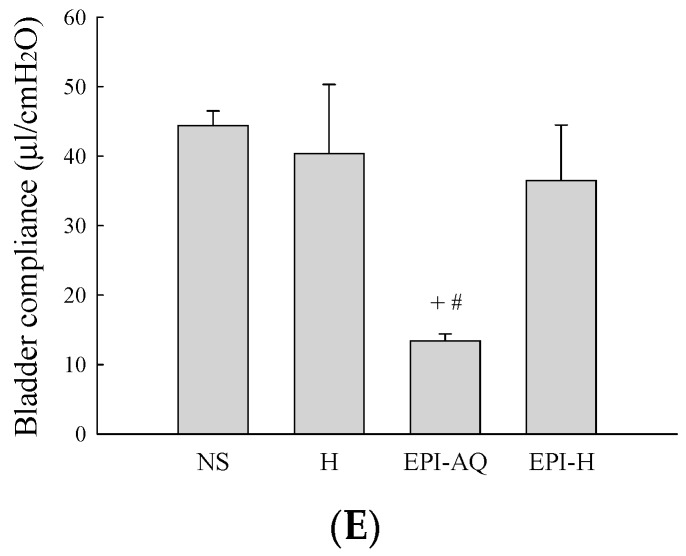
Effects of intravesical epirubicin on voiding function in the rat. (**A**) Representative tracings of cystometrograms (CMG) recorded from F344 rats following intravesical instillation of normal saline (NS), H, EPI-AQ, and EPI-H. Urodynamic parameters after treatments were (**B**) peak micturition pressure (cmH_2_O); (**C**) threshold pressure (cmH_2_O); (**D**) micturition duration (s); and (**E**) bladder compliance (μL/cmH_2_O). Data are mean ± S.D. ^+^ *p* < 0.05 compared with NS. ^#^ *p* < 0.05 compared with H. * *p* < 0.05 compared with EPI-AQ.

Intravesical instillation of epirubicin to patients causes severe bladder irritation and induces early voiding. Thus, understanding the urodynamic effects of EPI-H is crucial for assessing its potential for future clinical application. CMG profiles following intravesical instillation of normal saline (NS), H, EPI-AQ, and EPI-H in rats was recorded (Figure 4A). The peak micturition pressures in NS, H, EPI-AQ, and EPI-H treatment groups were 21.2 ± 0.3, 18.7 ± 0.1, 51.0 ± 1.8, and 31.1 ± 1.7 cmH_2_O, respectively (Figure 4B). EPI-AQ elicited the highest peak micturition pressure, and EPI-H induced less bladder hyperactivity. The threshold pressures observed in NS, H, EPI-AQ, and EPI-H treatment groups were 1.6 ± 0.1, 1.8 ± 0.4, 5.3 ± 0.4, and 2.0 ± 0.5 cmH_2_O, respectively (Figure 4C). EPI-H treatment also significantly reduced threshold pressure when compared with the treatment effect of EPI-AQ.

Bladder capacity was measured as the amount of saline infused into the bladder at the time micturition commenced. The infusion durations observed in the NS, H, EPI-AQ, and EPI-H treatment groups were 445.0 ± 5.0, 313.3 ± 10.6, 309.2 ± 4.7, and 214.0 ± 3.0 s, respectively (Figure 4D). The bladder compliances of the NS, H, EPI-AQ, and EPI-H treatment groups were 44.4 ± 2.6, 40.4 ± 12.1, 13.4 ± 1.2, and 36.5 ± 9.1 μL/cmH_2_O, respectively (Figure 4E). EPI-AQ treatment appreciably reduced bladder compliance (*p* < 0.05) when compared with the treatment effects of NS and H. In contrast, EPI-H treatment elicited bladder compliance results similar to that observed with the NS and H treatments. Overall, these observations demonstrated that EPI-H infusion diminishes epirubicin-induced bladder hyperactivity by reducing micturition peak and threshold pressure, lessening bladder capacity, and increasing bladder compliance.

### 2.5. In Situ Urothelium Penetration

**Figure 5:**
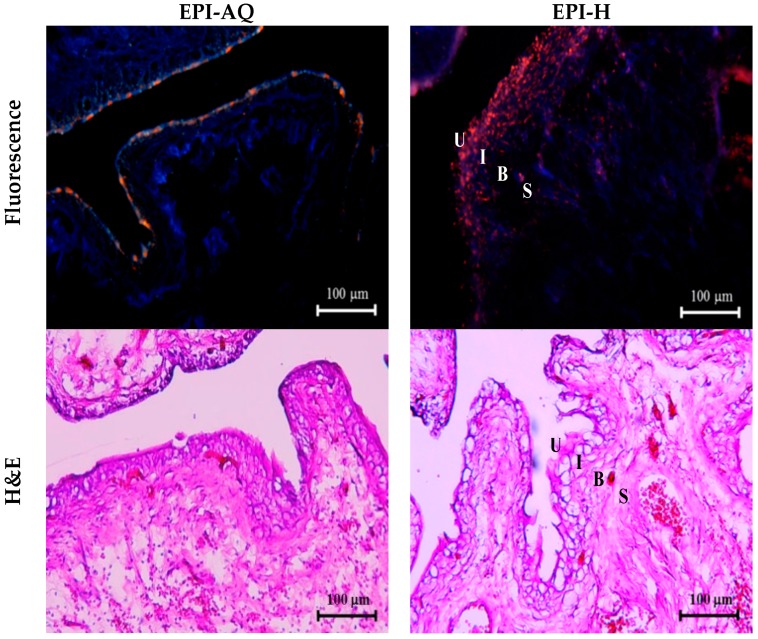
EPI-H enhanced penetration of epirubicin to urothelium. Fluorescence images of urothelium showed epirubicin (red) and DAPI (nuclei, blue) after intravesical EPI-AQ or EPI-H instillation in F344 rats. Histopathology sections were stained by hematoxylin/eosin (H&E). Umbrella cells, intermediate cells, basal cells, and subepithelial connective tissue of the urothelium are respectively indicated by U, I, B, and S. (magnification, ×200)

EPI-H and EPI-AQ were each intravesically instilled into rat bladder and epirubicin penetration into the urothelium associated with each treatment was measured. Epirubicin excites and emits as red under fluorescence microscopy. As shown in Figure 5, EPI-AQ, which is the form used in the reported clinical intravesical instillations, was present at extremely low levels in the superficial plaque layer of the bladder. EPI-H forms a dense coating (in red) on the urothelial surface, which indicated that more epirubicin penetrates the plaques and that the epirubicin is distributed to umbrella cells, Tb cells, and basal cells above the lamina propria.

Histological assessment following a 2-h instillation with EPI-AQ revealed that the urothelium of the urinary bladder was intact, with rounded surfaces of the umbrella cells, and showed slight submucosal edema and inflammation. With instillation of EPI-H, the umbrella and intermediate cells of the urothelium exhibited a mild loss of integrity at focal areas where the epithelium showed some subtle denudation. EPI-H showed detrimental effects with signs of cell damage, inflammation and epithelium denudation. Histological staining revealed no remarkable microscopic features in the suburothelial stroma.

**Figure 6:**
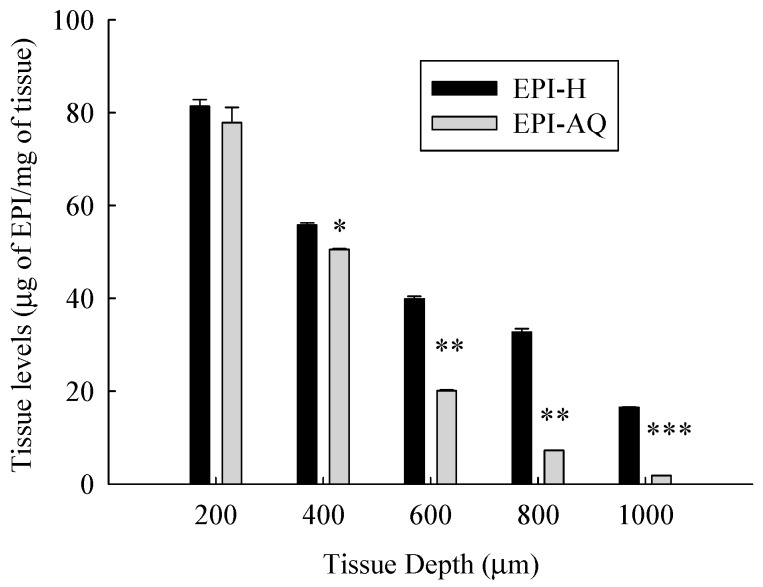
Tissue level-depth profiles of epirubicin in bladder tissue following exposure to 2 mg/mL EPI-AQ or EPI-H formulations. Tissues were incubated for 2 h and sectioned by cryotome. Values are means ± SEM (*n* = 3). * *p* < 0.05, ** *p* < 0.01 and *** *p* < 0.001 in cases of EPI-AQ *vs.* EPI-H.

Figure 6 presents the tissue concentration-depth profile of EPI-AQ and EPI-H in the pig bladder wall following the Franz diffusion assay. The concentrations of epirubicin were normalized according to the weight of wet tissue. 

The mean concentrations of epirubicin (μg/mg tissue) at EPI-H/EPI-AQ groups at a depth from the apical urothelium were 81.4/77.9 (at 200 μm), 55.8/50.5 (at 400 μm, *p* < 0.05), 39.9/20.1 (at 600 μm, *p* < 0.01), 32.7/7.3 (at 800 μm, *p* < 0.01), and 16.5/1.8 (at 1000 μm, *p* < 0.01), respectively. Tissue concentrations of epirubicin decreased as the tissue depth from the urothelium increased. EPI-H released more epirubicin into urothelium over that of EPI-AQ.

### 2.6. In Vivo Antitumor Efficacy

**Figure 7:**
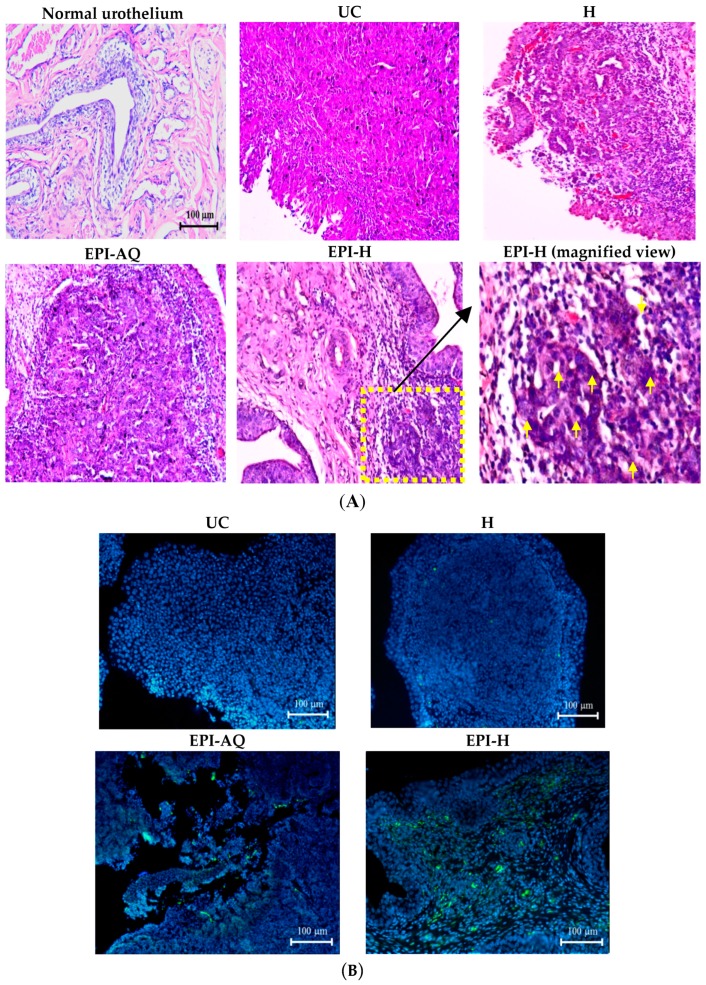

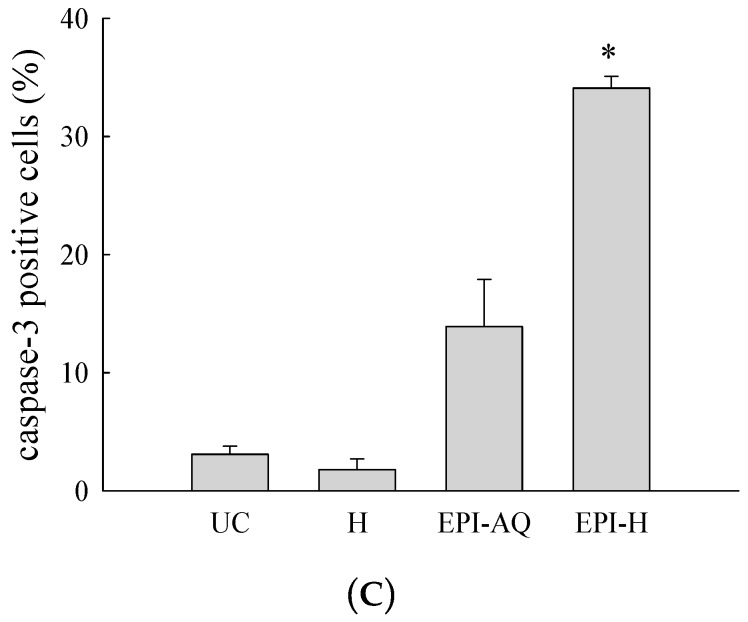
EPI-H enhanced antitumor efficacy in F344/AY-27 rat model. (**A**) Histopathologic findings with H&E staining showed normal urothelium; urothelium carcinoma (UC), and UC treated with H, EPI-AQ, EPI-H, and the enlarged image of yellow box in EPI-H. The yellow arrowheads indicated apoptotic nuclei. (**B**) Immunofluorescence of bladder section showed caspase-3 (green) and DAPI (nuclei, blue) in UC, H, EPI-AQ, and EPI-H groups. (magnification, 200×) (**C**) Quantitative analysis of caspase-3 in immunofluorescence images. Average of the positively stained area was evaluated from three randomly selected observation fields in each bladder section. * *p* < 0.001, compared with the EPI-AQ group.

The potential anti-cancer activity of EPI-H against AY-27 bladder tumors in F344 rats was evaluated. Normal (control) rat bladder showed an intact urothelium, including surface umbrella cells with scalloped appearance and fuzzy superficial cytoplasm (Figure 7A). AY-27 administration caused bladder tumor invasion from the urothelium to the muscle layer (T2 stage). The tumor cells exhibited an increase in the nuclear-to-cytoplasmic (N/C) ratio with markedly increased variation in nuclear size and shape, and mitotic figures. The hydrogel treatment group (H) also showed similar histologic features associated with bladder tumor including marked nuclear pleomorphism. In the EPI-AQ treatment group, low to moderate dysplasia and concentration of inflammatory cells were evident (T1 stage). Intravesical instillation of EPI-H however restricted tumor growth to the urothelium layer, prevented progress to advanced stages, and elicited necrosis of tumor cells. EPI-H showed detrimental effects with signs of cell damage, inflammation and epithelium denudation. The micro-features of the EPI-H treatment group showed marked inflammatory cell infiltration (e.g., lymphocytes and neutrophils) in the tumor. Several tumor nests were apparent with cell debris confined inside. These observations demonstrated the anti-cancer activity of EPI-H treatment against bladder tumors.

Levels of caspase-3 proteins were assessed by immunofluorescence microscopy of paraffin-embedded AY-27 tumor sections of F344 rats as a means to evaluate apoptosis (Figure 7B). EPI-H treatment induced higher caspase-3 levels than that induced by EPI-AQ treatment. These results confirmed that the increased apoptosis induced by EPI-H in AY-27 cells occurred through the caspase-3 activation pathway. The percentage of caspase-3-positive cells in the urothelium carcinoma (UC), hydrogel (H), EPI-AQ, and EPI-H groups were 3.1% ± 1.0%, 1.8% ± 0.9%, 13.9% ± 4.0%, and 34.1% ± 1.0%, respectively (Figure 7C). These results were consistent with the *in vitro* study results shown in Figure 3.

## 3. Discussion

This study examined the optimal epirubicin delivery system for intravesical therapy of bladder cancer as a means to improve drug efficacy and to reduce adverse effects in a rat tumor model. The response rate for intravesical drug instillation is approximately 30% when tumors are present, and approximately 70% after tumor surgery, and it differs according to the agent used [9]. Major limitations of intravesical chemotherapeutic drugs are attributable to low bladder residency and low penetration into the urothelium. This study showed, to our knowledge, for the first time the feasibility of gelatin hydrogel carriers as a means to administer intravesical epirubicin to treat more effectively superficial bladder cancer.

The advantage of intravesical chemotherapy is its restriction to the bladder and insignificant systemic drug absorption and distribution. The plasma uptake of any drug administered intravesically is dependent on its molecular weight (MW), and to a lesser degree, on its lipid solubility. Systemic absorption from the bladder of drugs with MW > 300 is minimal [10]. Absorption of epirubicin (MW 342) instilled into the bladder is generally slight, and plasma levels do not exceed 0.5 ng/mL [11]. In this study, intravesical instillation of EPI-H resulted in more epirubicin retention in the urothelium than that achieved by EPI-AQ instillation *in vivo* in an orthotopic rat model. Using the hydrogel carrier, more epirubicin penetrated plaques and distributed to umbrella cells, intermediate cells, and basal cells. Epirubicin reached the lamina propria but did not significantly penetrate muscle or bladder serosa. These experiments indicated that gelatin hydrogel carriers are suitable for applications in superficial urothelium cancer, a health problem providing a major indication for intravesical (rather than systemic) chemotherapy. The efficacy of EPI-H was also observed through histological measurements. EPI-H induced tumor cell necrosis and prevented tumor progression in a 2-week instillation protocol.

Gelatin transforms from gel to solution (sol) by raising the temperature. The reversible melting and congelation of gelatin, namely, solation and gelation, take place by heating and cooling, respectively. Gelated gelatin contains three straight chain molecules that combine to form complicated three dimensional structures. Heating of gelatin causes the disintegration of these three-dimensional structures followed by the dispersion of the chain molecules [12,13]. In present study, there is gel-sol transition at 25 °C. The aim of present hydrogel is applied for intravesical chemotherapy, thus, solution status in body temperature provide suitable fluidity to cover and adherent urothelium.

The loading efficiency of gelatin varies according to preparation methods and drug characteristics. A loading efficiency of 29%–32% was obtained by means of a water/oil emulsion method for methotrexate in gelatin nanoparticles [14]. A 15%–19% loading efficiency was reported for chloroquine phosphate hydrogel using a similar protocol [15]. The gelatin hydrogels produced in the present study were covalently crosslinked by glutaraldehyde, which served to increase stability and mechanical properties [16]. Glutaraldehyde is indispensable for tizanidine hydrochloride-loading; loading is 13.8% with glutaraldehyde-based preparation but only 1.1% without glutaraldehyde-mediated crosslinking [17]. Loading efficiency is also influenced by physiochemical properties of the drugs and of the gelatin concentration. In the present study, the epirubicin loading efficiency in 5% and 15% gelatin was 47.40% and 57.87%, respectively. 

The major adverse effects of epirubicin instillation are bladder irritation, hematuria, pyuria, atrophic bladder, and hemorrhagic cystitis [18]. Weekly instillations of epirubicin were reported to induce changes in vesical volume and compliance, although only the change in bladder compliance was associated with statistical significance [11]. Decreased bladder compliance is probably due to direct superficial epithelial damage and cell loss, leading to mucosal irritation and submucosal edema. In humans, chemical cystitis after intravesical chemotherapy manifests primarily as urinary frequency, urgency, and/or bladder pain. In this study, we determined whether functional bladder damage occurred after intravesical chemotherapy with EPI-H. Higher levels of epirubicin entering the urothelium would seem to inevitably lead increase the risk of chemical cystitis. In comparison with EPI-AQ treatment, the use of EPI-H resulted in lower micturition duration, indicating decrease bladder capacity. Fortunately, the bladder compliance was significantly higher in the EPI-H treatment group when compared with that of the EPI-AQ treatment group.

Flow cytometry analysis showed that EPI-H treatment doubled the number of AY-27 cells entering the early stages of apoptosis. We determined that this was related to an increased cleaved-/pro-caspase-3 ratio in the treated AY-27 cells. Caspase-3 expression in the AY-27 rat bladder cancer cell line is known to be associated with the apoptotic response [19]. Caspase-3 is also involved in bladder cancer apoptosis when cells are exposed to epirubicin [20]. Apoptosis failure is a crucial step in the initiation and progression of cancer. This information can be used clinically to select chemotherapeutic drugs that act in a hormone-dependent or independent manner. For therapeutic intravesical chemotherapy, bladder cancer cells are more sensitive to EPI-H than to the currently used EPI-AQ.

Intravesical EPI-H instillation exhibited an anti-cancer effect on a urothelium carcinoma that had been orthotopically implanted into a rat model. An orthotopic bladder tumor model is indispensable in studies of therapeutic effects. AY-27 rat bladder cancer cells transplanted orthotopically into Fischer F344 female rats exhibit features of transitional cell carcinoma (TCC) [21]. Patchy carcinoma *in situ* can be detected histologically at 12–13 days after inoculation, and the condition progresses to papillary tumor or invasive disease thereafter. An NMIBC study indicated that the 2-year recurrence-free survival rate following epirubicin treatment is 55% [22]. Intravesical epirubicin in the NMIBC study resulted in a recurrence rate of 36%–62%. There were 14–38 months in median time to recurrence, and the 5-year cumulative recurrence rate was 58%–69% [1]. The presently developed EPI-H system has an improved intravesical chemotherapeutic ability to prevent massive tumor progression and invasion, and it could improve the numbers described above.

## 4. Materials and Methods

### 4.1. Materials

Epirubicin was obtained from Calbiochem (La Jolla, CA, USA). Gelatin, glycine, propidium iodide (PI), and glutaraldehyde were purchased from Sigma Chemical Co. (St. Louis, MO, USA). All organic solvents were of HPLC grade. All cell culture media and reagents were from Gibco BRL (Grand Island, NY, USA) or Hyclone (Logan, UT, USA). The annexin V-FITC kit was purchased from Calbiochem. Fischer F344 rats were purchased from the National Laboratory Animal Center (NLAC) (Taipei, Taiwan). Animal protocols were approved by the Animal Ethics Committee of I-Shou University.

### 4.2. HPLC Analysis of Epirubicin

Epirubicin was analyzed by HPLC with fluorescence detection (FLD) [7]. Briefly, HPLC-FLD analyses were carried out using a model L7100 solvent delivery system (Hitachi, Tokyo, Japan). An AcclaimR120 (150 mm × 4.6 mm ID) (Dionex, Surrey, UK) column was used for separation of EPI. The mobile phase consisted of acetonitrile/0.1 M orthophosphoric acid/triethylamine/water at volumetric ratios 27:3:0.07:70 with an apparent pH of 2.8. The flow rate of the mobile phase was 1.0 mL/min. Doxorubicin was used as an internal standard. The column outlet stream was monitored at λ_ex_-474 nm and λ_em_-551 nm. Linearity was found to be in the range of 0.2–40 μg/mL with a coefficient of determination (R^2^) of 0.9999. The limit of detection (LOD) and limit of quantitation (LOQ) were 0.02 and 0.07 μg/mL, respectively.

### 4.3. Preparation of Gelatin Hydrogel

Hydrogel was prepared according to previous methods [8] using A type gelatin (Sigma) with 75 or 175 bloom numbers. An aqueous solution of 5% (*w*/*v*) gelatin (1 mL) with 0.8 μg/mL of glutaraldehyde was left at 4 °C overnight for gelation and cross-linking action. The cross-linked gelatin hydrogel was immersed in 50 mM glycine aqueous solution under agitation for 1 h to block the residual aldehyde groups of glutaraldehyde, followed by washing twice in double-distilled water for 1 h. The resulting hydrogel was freeze-dried for 48 h. Freeze-dried gelatin was dissolved in 1 mL of normal saline to form 15% (*w*/*v*) hydrogel, and the solution was heated to 37 °C.

### 4.4. Characterization of Hydrogel

After allowing the freeze-dried hydrogel to swell for 24 h at 37 °C in normal saline, the weight of swollen hydrogel (Ws) was measured. The swollen hydrogel was dried in a vacuum drying oven at 60 °C for 6 h, and then the weight of vacuum-dried hydrogel (Wd) was measured. The hydration ratio was calculated using the following equation: [(Ws − Wd)/Ws] × 100. The viscosity of hydrogel was determined using a Carri-Med CSL2 100 rheometer (TA Instruments, New Castle, DE, USA). Particle size distribution and mean diameter were determined using an N5 Submicron Particle Size Analyzer (Beckman, Hialeah, FL, USA). The surface morphology of freeze-dried hydrogel was determined using a field emission scanning electron microscope (FE-SEM; JEOL JSM-5600 LV, Tokyo, Japan). Hydrogel was frozen in liquid nitrogen prior to freeze-drying to maintain the existing morphology. Sectioned gels were mounted on metal holders and vacuum-coated with a gold layer prior to SEM examination. The fluidity of the hydrogel was visually monitored at 25 °C and 37 °C [8].

### 4.5. Entrapment Efficiency of EPI-H

Epirubicin was distributed in 15% (*w*/*v*) hydrogels for the following physicochemical properties and bioactivity evaluation. To determine the entrapment efficiency, EPI-H hydrogel (0.1 g) was dissolved in 10 mL of normal saline, centrifuged and the epirubicin content of supernatant was determined by the aforementioned HPLC method:

$$\mathrm{entrapment}\mathrm{efficiency}(\%)=\frac{\mathrm{actual}\mathrm{epirubicin}\mathrm{concentration}}{\mathrm{theoretical}\mathrm{epirubicin}\mathrm{concentration}}\times 100$$

### 4.6. Cell Culture and Cytotoxic Assay

The rat bladder cancer cell line AY-27 (a kind gift from Professor R. Moore, University of Alberta, Edmonton, AB, Canada) was cultured in RPMI-1640 medium supplemented with 10% fetal bovine serum (FBS), 2% L-glutamine, and 0.2% penicillin/streptomycin at 37 °C with 5% CO_2_ [21]. Cell viability was determined using the CellTiter 96 Aqueous nonradioactive cell proliferation assay according to the manufacturer’s instructions (Promega, Madison, WI, USA) [23]. AY-27 cells (1 × 10^4^) were seeded in 96-well plates and then treated with epirubicin-loaded hydrogels for 18 h. Cell-growth inhibition potency was expressed as IC_50_ values, defined as the concentration of the drug necessary to inhibit the growth of cells by 50% in 18 h. The percentage of surviving cells was defined as (mean absorbance of treated wells/mean absorbance of untreated wells) × 100%. Data are the means ± S.D. of four experiments. 

### 4.7. Flow Cytometry with Annexin V-FITC and PI Staining

AY-27 cells were pretreated with EPI-H or EPI-AQ (as final concentration of epirubicin 0.25 μg/mL) for 18 h. AY-27 cells were harvested, washed with ice-cold phosphate buffered saline (PBS), and stained with Annexin V-FITC (AV)/PI reagent as described previously [24]. Apoptosis rates were quantified by means of fluorescence-activated cell sorting flow cytometry (BD Biosciences, Ann Arbor, MI, USA). AV-positive/PI-negative cells were scored as early apoptotic, and AV-positive/PI-positive cells were counted as late apoptotic cells.

### 4.8. Western Blotting

AY-27 cells were pretreated with EPI-H or EPI-AQ (as final concentration of epirubicin 0.25 (μg/mL) for 18 h. AY-27 cells were washed twice in cold PBS, and lysed in TBS containing 1 mM EDTA, 1 mM DTT, 0.2% Triton, 0.1% SDS, and the complete protease inhibitors mixture (Roche, Indianapolis, IN, USA). Proteins were subjected to SDS-PAGE, and then transferred to polyvinylidene difluoride membranes (Boehringer Mannheim, Indianapolis, IN, USA). Membranes were probed with antibodies specific for caspase-3 (Cell Signaling, Danvers, MA, USA), cleaved caspase-3 (Cell Signaling), or actin (Merck Millipore, Darmstadt, Germany), and incubated with the HRP-conjugated anti-rabbit Ig or anti-mouse Ig Ab. Signals were revealed with the ECL kit (Merck Millipore), and visualized by autoradiography. Increased levels of cleaved-caspase-3 (17 kDa) with a concomitant decrease in levels pro-caspase-3 (32 kDa) is regarded as an indicator of apoptosis.

### 4.9. Cystometrogram (CMG) Analysis

The CMGs were carried out according to previously described methods [25,26]. Fischer F344 rats (7 weeks) were anesthetized with Zoletil-50 (1 mg/kg). Before the beginning of each CMG, the bladder was emptied, and a urethral catheter was inserted to fill the bladder and to measure bladder pressure. The catheter was connected via a T-tube to a syringe pump (KDS250, KD Scientific Corp., MA, USA) and to a pressure transducer and amplifier (ML866 and ML224, PowerLab, AD Instruments, Colorado Springs, CO, USA). Data were recorded on a chart recorder and digitized for computer data collection (Labchart 7, AD Instruments, Colorado Springs, CO, USA). The bladder was then infused with 500 μL normal saline (as control), and epirubicin-loaded hydrogel at a steady rate (0.07 mL/min), during which time the pressure was measured in-line through the catheter. A voiding contraction was defined as an increase in bladder pressure that resulted in urine loss. CMG was recorded until the bladder pressure was stable, and at least 5 filling/voiding cycles were measured on each rat before drug administration, and used as baseline values. CMG parameters recorded for each animal included peak micturition pressure, threshold pressure, duration of non-voiding contractions (without urine leakage during bladder infusion), and bladder compliance. Peak micturition pressure was the maximum pressure during micturition as observed in CMG. Threshold pressure was the intravesical pressure immediately prior the initiation of micturition. Bladder compliance was measured by infused volume (μL)/threshold pressure (ΔcmH_2_O).

### 4.10. Ethics Statement

All in-life rat studies were performed under Association for Assessment and Accreditation of Laboratory Animal Care-approved conditions and approved by the I-Shou University Institutional Animal Care and Use Committee (Approval numbers IACUC-ISU-102025). Animals were sacrificed by carbon dioxide asphyxiation.

### 4.11. In Vivo Urothelium Permeability and Histologic Analysis

Female F344 rats (7 weeks) were treated with EPI-H or EPI-AQ (500 μL/180 g b.w.) for 2 h via urethral catheter. After administration, rat urinary bladder tissue pieces with flat surfaces were sectioned by CM 1900 cryostat (Leica, Amtzell Germany) and observed by fluorescence microscope (Eclipse 4000, Nikon, Tokyo, Japan). The remaining rat bladder tissue was fixed in formalin, embedded in paraffin, and stained with hematoxylin and eosin (H&E) for histological analysis [27].

### 4.12. Determination of Epirubicin in the Bladder Tissues

Fat removed, freshly excised porcine bladders were opened and cut into pieces measuring approximately 3 × 3 cm in a shallow bath of pH 7.4 PBS buffer. Bladder sections were mounted onto a Franz diffusion cell apparatus, such that the luminal side of the bladder wall faced upwards to contact the drug solution as previously described. The tissue sections were not stretched and measured approximately 2–3 mm thick. Receptor chambers contained 6 mL of 37 °C PBS buffer (pH 7.4). The donor chambers contained 0.5 mL of EPI-H and EPI-AQ (all 2 mg/mL drug). The diffusion cells were maintained at 37 °C for 2 h. The drug contact areas of the tissue samples were then cut out and rapidly frozen with liquid nitrogen on a bed of dry ice. Tissues were then sectioned into 200 μm thick samples by cryostat (Leica CM 1900). Epirubicin concentrations in the tissue were determined by HPLC [7].

### 4.13. Bladder Tumor Implantation, Intravesical Drug Instillation and Histology Analysis

In situ urothelium cancer was induced by intravesical instillation of the rat AY-27 bladder tumor cell line [21]. F344 rats were anaesthetized, and the bladder was catheterized via urethral catheter. To facilitate tumor seeding, the bladder mucosa was conditioned with 0.4 mL of 0.1 N hydrochloric acid (HCl) for 15 s and neutralized with 0.4 mL of 0.1 N potassium hydroxide (KOH) for 15 s. The bladder was then drained and flushed with sterile PBS. Immediately after bladder conditioning, the AY-27 cells ((1 × 10^7^) were instilled and left indwelling for at least 1 h. The rats were turned 90° every 15 min to facilitate whole bladder exposure to the tumor cell suspension. 

After 1 h, the catheter was removed and the rats were allowed to void the suspension spontaneously. After tumor implantation (on 1st day), F344 rat bladders were intravesically instilled with EPI-H on the 8th, 10th, and 12th days. All of the rats were killed on the 14th day. After killing, bladders were excised, fixed in 4% formalin overnight, dehydrated, and then embedded in paraffin. The embedded tissues were cut into 5-μm slices and stained with hematoxylin and eosin (H&E) for histological analysis.

### 4.14. Urothelium Caspase-3 Immunofluorescent Staining

Paraffin-embedded tumor sections were subjected to immunofluorescence analysis. Briefly, sections were deparaffinized, rehydrated, subjected to heat-induced antigen retrieval, and quenched. Sections were blocked with Antibody Diluent (Dako, Carpinteria, CA, USA) and incubated with a caspase-3 antibody (1:500) (Cell Signaling). Sections were then washed and incubated with anti-rabbit IgG and counterstained with mounting media containing DAPI and analyzed using a microscope (Eclipse 4000, Nikon). The number of caspase-3+ cells in the bladder sections was counted in 3 randomly selected fields (×200), and the average of the 3 fields was calculated [28].

### 4.15. Statistical Analysis

The data are presented as mean ± standard deviation (SD). Data were analyzed using the student *t*-test and one-way analysis of variance (ANOVA). Statistical significance was determined at the level of *p* < 0.05.

## 5. Conclusions

Gelatin hydrogels are effective carriers of epirubicin for intravesical chemotherapy of superficial urothelium cancer. The strong bio-adhesive property of gelatin hydrogel minimizes flushing away by urine, thereby enhancing antitumor efficacy of loaded drugs. Hydrogel delivery of epirubicin represents an attractive strategy for intravesical drug development.

## Figures and Tables

**Table 1:** Characterization of gelatin hydrogel.

Gelatin	Yield (%)	Hydration Ratio (%)	Size (nm)	Viscosity (Pa.s)	Torque (%)
A75	89.7 ± 21.0	86.8 ± 0.0	183.6 ± 4.6	32.2 ± 1.3	56.7 ± 6.6
A175	98.0 ± 24.9	87.2 ± 0.1	124.6 ± 0.9	78.7 ± 3.0	67.0 ± 4.5
